# Critical Pollination Chemistry: Specific Sesquiterpene Floral Volatiles in Carrot Inhibit Honey Bee Feeding

**DOI:** 10.1021/acs.jafc.3c03392

**Published:** 2023-10-23

**Authors:** Stephen R. Quarrell, Alyssa M. Weinstein, Lea Hannah, Nicole Bonavia, Oscar del Borrello, Gavin R. Flematti, Björn Bohman

**Affiliations:** † Tasmanian Institute of Agriculture, University of Tasmania, College Rd, Hobart 7005, Australia; ‡ Ecology and Evolution, Research School of Biology, The Australian National University, Canberra 2601, Australia; § Seed Production Research, Research and Development, Rijk Zwaan Australia, Musk, Victoria 3461, Australia; ∥ Hawkesbury Institute for the Environment, Western Sydney University, Richmond, New South Wales 2753, Australia; ⊥ School of Molecular Sciences, University of Western Australia, Crawley, Western Australia 6009, Australia; # Department of Plant Protection Biology, Swedish University of Agricultural Sciences, Lomma 234 22, Sweden

**Keywords:** pollination, chemistry, crop, sesquiterpene, floral volatiles

## Abstract

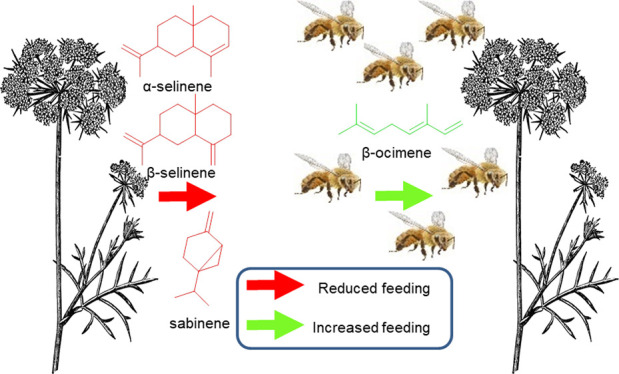

Many plants rely on insect pollination, yet numerous agricultural plant-breeding programs focus on traits that appeal to growers and consumers instead of pollinators, leading to declining pollinator attraction and crop yields. Using hybrid carrot seed production as a model, we investigated low-yielding carrot varieties by analyzing sugars and minerals in nectar and floral volatile composition. While the analysis of nectar sugars and minerals did not reveal any key differences between the carrot varieties, differences between the 112 detected volatiles in 23 samples were observed. Numerous differentiating sesquiterpenes were identified in floral solvent extracts, and subsequent behavioral assays showed that β-ocimene from higher-yielding carrot varieties stimulated nectar feeding (attractant), while α- and β-selinene from lower-yielding lines decreased feeding (deterrents). Sesquiterpenes have previously been implicated in plant defense, suggesting a trade-off between pollination and protection. Our results highlight the importance of volatiles as regulators of pollinator attraction in agricultural settings.

## Introduction

Ecosystem function is underpinned by numerous biotic and abiotic factors. In angiosperms, effective pollination is crucial for sexual reproduction and, in many instances, is facilitated by insect pollinators.^1^ When foraging, pollinators typically show floral preferences that are mediated by a suite of visual, olfactory, and gustatory cues.^2^ The key traits that attract pollinators are considered to include direct rewards such as nectar^3,4^ and pollen,^5,6^ attractive floral volatiles, and visual cues.^7,8^ However, cross-kingdom interactions can be complex, as social insects do not solely use direct cues of attraction when foraging and may heed or ignore resources based on a variety of other associative factors communicated by siblings. These decision-making processes may include other stimuli including the presence of predators,^9^ shifts in the prevalence of a specific resource, or potentially the perceived quality of a specific resource relative to others that become available within the foraging range.^10^ For such reasons, physical attributes of flowers alone do not necessarily trigger foraging behavior instantly and insect behavior can change once a particular cue has been encountered.^11^ Many pollinators display flexibility in their preferences due to associative learning between rewards and floral characteristics.^12^ The interaction between these cues can shift floral visitation from abundant resources toward higher-quality, less abundant forage. For example, many agricultural crops that have a periodic overabundance of floral resources may not always possess the nectar rewards or attractive floral cues required to induce pollinator visitation.^13,14^ Therefore, it is essential to consider insect learning processes to understand pollination efficiency, particularly within agricultural production systems.^15^

With over 35% of global food crops at least in part dependent on animal pollination,^16,17^ the foraging preferences of pollinators within agricultural crops is being increasingly recognized as an important factor in maintaining agricultural production levels and quality.^18^ Economically, it has been estimated that nearly 10% of the total value of agricultural production, or ∼US$200B is derived from insect pollination.^19^ Although many animals are important pollinators, the European honey bee *Apis mellifera* remains the most widely used pollinator for commercial crops.^16^ However, diseases, parasites such as the Varroa mite, use of pesticides, and other factors have led to a drastic decline in honey bee numbers in recent decades.^20−22^ Strategies to safeguard global food production in the face of drastic declines in honey bee numbers include an enhanced focus on alternative pollinators^23,24^ and revised crop management strategies.^25,26^ No doubt the question of securing pollination services, for both biological conservation and food production, deserves a broad focus. Nonetheless, there is also an immediate knowledge gap regarding the importance of chemical cues with respect to pollinator floral preferences for agricultural crops.^27^ First, despite the common use of managed honey bee hives in agricultural production systems, pollen transfer between flowers often remains limited, especially in crops that do not naturally depend on bees for pollination.^28−30^ By increasing hive density, crop pollination rate and subsequent seed set can be improved, but for some varieties, such measures remain insufficient with low yields still common.^31^ Second, several crops grown for human consumption are selectively bred for traits favored by growers and consumers, such as elevated pest and disease resistance and appealing taste and physical appearance, but are seldom bred for pollinator attraction.^32^ This trend has led to pollination deficits becoming increasingly noticeable, particularly with the introduction of hybrid crop varieties. Several modern crops experience reduced seed yields despite managed honey bee hives being used in excess.^33^

Honey bees are well-known to associate rewards with phenotypic cues such as color and floral volatiles.^15^ The influence of color and floral morphology in particular has been studied in detail for bee pollination (e.g., Menzel and Müller^34^ Giurfa et al.^35^). Yet, despite their importance particularly within vegetable seed production, the detailed cues underpinning honey bee attraction to vegetable flowers remain poorly understood. In flowering hybrid crops, bees may initially orient toward visual cues in the flowers of one cultivar or accession. Once these flowers are located, more attractive chemical cues from alternative neighboring cultivars or accessions could override the initial attractive signals. It has been established that although possible, it is difficult to train social insects by manipulating associative cues without linking these traits with a reward.^3,34,36^ What appears to be much less known is how and why certain floral traits, which appear not to be linked with rewards, are avoided. In such cases, for example, for olfactory cues, where the lack of attraction appears to be independent of the degree of reward, there may be scope to affect insect behavior by manipulating or reducing the trait, thereby improving pollinator attraction. The accepted importance of floral volatiles, however, has led to the development of pollination-improving methodologies relying on the conditioning of pollinators to odors that are abundant in the flowers of target crops. By manipulating the pollinators, often honey bees, to associate these odors with rewards, the yields can be improved, but not always.^37^ Apart from the costly and time-consuming process involved in pollinator preconditioning, the long-term efficiency of these methodologies is questionable, as the insects learn that the level of reward is not maintained.^37^

Pollinator attraction is often measured by quantifying pollination rates and improved seed yields.^38−40^ To attempt to decipher chemical attraction, detection of pollinator responses to individual selected chemical components of nectar or the floral headspace can be measured with antennal electrophysiology methods or honey bee proboscis extension response bioassays.^41−44^ However, routine electrophysiology methods cannot detect all biologically active compounds, nor distinguish between the different functions of the semiochemicals being assessed (e.g., if attractant, repellent, or other).^45^ Proboscis extension response bioassays suffer from a strong association between the response and reward, or in other words, responses are linked to memory effects,^45,46^ with any compounds that the bees have not been conditioned to potentially going undetected. Consequently, to observe the natural behavior of the pollinators, full behavioral bioassays are preferred.^47^ The importance of specific volatiles from crop flowers in honey bee attraction has previously been demonstrated in field bioassays with alfalfa using artificial flowers.^48^

Carrots (*Daucus carota* L.) are an important commercial crop, for which it has been established that pollination limitation is a major contributor to low seed yields^49^ and multiple studies have shown that the pollination rates of hybrid varieties are lower than in open-pollinated (OP) varieties.^50,51^ Compared to alternative pollinators,^52−56^ as for most crops, honey bees remain the most effective pollinators of hybrid carrots.^53^ Thus, the hybrid carrots represent a suitable model for investigating variables that negatively affect pollination success in honey bee-pollinated cropping systems. The effects of color and floral morphology have been studied for bee pollination of carrots, with inflorescence color and nectar sugar composition and concentration found not to differ significantly between hybrid lines.^57,58^ However, no comprehensive studies of the role of specific volatiles have been reported.

In this study, we compare the attractiveness of four parental carrot accessions including two sterile, pollen-free, cytoplasmic male sterile (CMS) carrot lines and their reciprocal fertile pollen-bearing, fertile “maintainer” lines. Each pair bears the same nuclear genome with the sterile CMS parent being emasculated via the genetic manipulation of the *restorer of fertility* (*R*_f_) genes within the plant’s cytoplasm.^59^ Industry-documented seed set data for each pairing indicated that each reciprocal pair (fertile and sterile) yielded either consistently low or moderate seed yields. These accessions were compared to a fifth OP cultivar, Western Red. The OP cultivar is known to produce high seed yields and is considered to be highly attractive to honey bees. We hypothesized that nectar from the carrot accessions producing less seed would be less attractive to honey bees and aimed to determine the factors contributing to differences in honey bee attraction to different carrot accessions. The traits of nectar composition (floral reward; sugar and micronutrient concentration and composition) and volatile composition (floral attraction) were investigated. In addition, bee attraction to individual characteristic chemical compounds of specific accessions was tested and confirmed in behavioral bioassays.

Analysis of the carrot nectars showed no difference in the sugar content or composition between carrot lines. Further, honey bees avoided feeders during both field- and laboratory-based bioassays containing nectar from all carrot lines, indicating a general nonattractant effect. Despite no difference in floral reward, certain compounds isolated from carrot flowers and nectar not only failed to elicit attraction but functioned as repellents, including the sesquiterpenes α-selinene and β-selinene, while others enhanced attraction, e.g., β-ocimene. Sesquiterpenes have previously been implicated in pollinator attraction, repellence, and plant defense suggesting a fine balance between pollination and plant protection, which when altered within plant breeding programs can inadvertently impact floral visitation and crop yield within agricultural settings.

## Materials and Methods

### Carrot Lines

**Figure 1:**
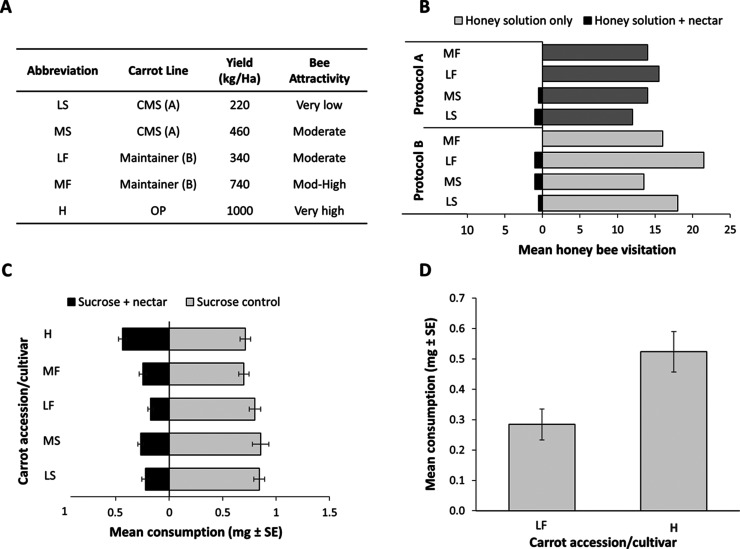
(A) Descriptions of four carrot accessions (LS, MS, LF, and MF) and one OP cultivar, Western Red (H) used in this study, with seed yields and relative honey bee attractivity. (B) Results from preliminary field bioassays. Number of bees feeding from each feeder. Controls consisted of honey solution, treatments of honey solution with added carrot nectar from each carrot parental accession (L and M) extracted with protocol A (dark gray bars) and protocol B (light gray bars). Controls were conducted pairwise with each treatment, with corresponding colored bars. (C) Results from laboratory bioassays and pairwise choice experiments comparing sucrose solutions with sucrose solutions spiked with carrot nectars for each accession and cultivar. (D) Results from laboratory bioassays and a pairwise choice experiment comparing sucrose solution spiked with nectar from line LF with sucrose solution spiked with nectar from variety H.

For the ongoing production of hybrid carrot seed, three parental lines are required: The A-line, (cytoplasmic male sterile line used as the female parent), the B-line is the “maintainer” line (male parent to produce more female A-line), and a third R-line (restorer), which is the pollinator or fertile male parent, which is crossed with the A-line to produce hybrid seed.^59^ Carrot seed from the two reciprocal pairs of petaloid, parental male sterile (LS = low-yielding sterile or MS = medium-yielding sterile) and male fertile (LF = low-yielding fertile or MF = medium-yielding fertile), carrot accessions (Figure 1A) were supplied by seed company Rijk Zwaan Australia Pty Ltd. Industry-documented seed set data for each pairing indicated each reciprocal pair (fertile and sterile) yielded either consistently low or moderate seed yields. Carrot seed from a fifth, high-yielding, OP cultivar, Western Red (H), was purchased from commercial seed supplier “The Diggers Club” (https://www.diggers.com.au/). All seed was planted in 15 cm plastic plant pots using a commercially sourced potting mix and housed in a glasshouse at 23 °C. Once fully grown, the carrot stecklings were vernalized to induce flowering by removing them from the pots, with any excess soil removed by washing them in tap water. The stecklings were then dipped in the fungicide Mancozeb Plus (Yates, Product code: 53850) and buried in polystyrene boxes filled with moist river sand and refrigerated at 4 °C for 10 weeks. Once vernalized, the carrots were repotted into 20 cm pots using the same commercially available potting mix and grown outdoors to flowering. For all four parental lines and the OP cultivar, umbellets were picked from the first, second, and third umbels between 10 am and 2 pm, 3–6 days after the opening of the first flower of each umbel. Samples were processed within 1 h of collection.

### Nectar Extraction for Bioassays and Chemical Analysis

For bioassays, carrot nectar was extracted following one of the following protocols. (A) Spinning in Eppendorf tubes, in a modified method from Giralamo.^60^ A spin filter (UltraClean Mini Plasmid Prep, Mo Bio Laboratories Inc., USA) in an Eppendorf tube was fully packed with individually collected carrot umbellets, with all stalks removed. Each sample was spun at 12,000 rpm for 10 min at room temperature. The carrot tissue was removed, and the filter was repacked with fresh umbellets and respun. The procedure was repeated until ca. 200 μL of nectar was separated. (B) To rule out any risk of contamination by coextracted sap, not naturally accessible to the pollinators, method B, an alternative method without mechanical extraction, was also applied. In this semiquantitative method, umbellets (*n* = 60) were individually dipped in water (2 mL), 20 times per umbellet, forming an extract of ca. 200 μL. Nectar samples from both methods were stored at −20 °C until used in bioassays. For chemical analysis, nectar was extracted by protocol B with minor modifications: Umbellets (*n* = 20) were dipped, one by one, 10 times in distilled water (1.0 mL). To the aqueous extract (ca. 100 μL) in an Eppendorf tube, dichloromethane was added (100 μL). The sample was vortexed (10 s), and an aliquot (50 μL) of the organic layer was removed for the analysis of volatiles. The remaining sample was concentrated under nitrogen and taken up in distilled water (100 μL). An aliquot (10 μL) was removed for carbohydrate analysis, while the remainder was used for atomic emission spectroscopy (AES) analysis.

### Initial Field Bioassays

The bioassay setup consisted of feeders made from clear plastic specimen jars of 5 cm diameter, with blue lids on which Eppendorf lids were glued upside down. The treatments were placed in these Eppendorf lids, which acted as dispensers. The feeders were placed on a fence ca. 1 m from the ground, intercepting a grassy slope approximately perpendicular and 20 m away from two managed beehives (ca. 30,000 bees per hive) at Sandy Bay, Tasmania. First, we confirmed that pure carrot nectar was not attractive to the honey bees within 4 h, even after several attempts to train the honey bees to the feeders from nearby hives. Then, we conducted sequential experiments with volumes of 50 μL per test in the following order: (1) honey solution (20% sugar); (2) treatment (nectar extracted with method A or B, above); (3) honey solution (20% sugar); (4) treatment spiked with honey (3:1 treatment to honey solution); and (5) honey solution (20% sugar). All experiments were conducted in duplicate on different days. As a control experiment, honey solutions were diluted four times to exclude the variable sugar content as the cause of lack of feeding in the treatments. Diluted honey (5% sugar) still promoted feeding.

### Laboratory Bioassays

All laboratory bioassays were conducted in a controlled temperature room at the University of Tasmania. The room conditions were 30–34 °C, with >50% humidity and an 8L:16D lighting regime. Bug Dorm (MegaView Science, Taiwan) cages, 25 × 25 × 25 cm were used with 10 foraging honey bees housed in each cage. Nectar-foraging bees were collected from the hive entrances at the University of Tasmania’s research apiary, Sandy Bay, Tasmania. All bees were collected while leaving the hive and transported to the controlled temperature room within 30 min of collection. Bees from each cage were sourced from one hive only. Upon arrival, each cage was provided with 40% w/w sucrose solution from a feeder consisting of two Eppendorf tubes (2 mL) suspended ca. 0.5 cm from the cage floor in an Eppendorf tube holder with the tubes spaced 10 cm apart. Each Eppendorf tube had 3 × 1.5 mm holes drilled into the terminal end to allow bee feeding. All experiments commenced upon arrival during the room’s photophase and lasted 48 h. The first 24 h allowed the bees to both acclimatize to the bioassay conditions and ensure no positional bias occurred regarding feeding tube preference. No positional bias was observed in any of the bioassays conducted after the initial 24 h (*P* > 0.05). The treatment period commenced with the Eppendorf tubes being replaced with either a tube filled with 40% w/w sucrose solution spiked with treatment solution or sucrose alone (control). Taking advantage of the partial water-solubility of secondary carrot metabolites, the treatment emulsions of such organic compounds in aqueous media were used as approximate mimics of the natural flower. The bee feeding from each tube was quantified by weighing the tubes before and after each bioassay. Results from any experiments with bee mortality exceeding 20% were discarded. For all laboratory bioassay experiments (see Table 1), data were analyzed by either a Wilcoxon signed rank test or paired *t* test depending on the outcomes of assumptions testing (Shapiro–Wilk) using SPSS version 26 (IBM Corp., Armonk, N.Y., USA).

**Table 1:** Laboratory Bioassays Conducted

**experiment**	**choice 1**	**choice 2**
1	sucrose solution	sucrose solution + LS
2	sucrose solution	sucrose solution + MS
3	sucrose solution	sucrose solution + LF
4	sucrose solution	sucrose solution + MF
5	sucrose solution	sucrose solution + H
6	sucrose solution + L_F_	sucrose solution + H
7	sucrose solution	sucrose solution + β-ocimene (**1**)
8	sucrose solution	sucrose solution + sabinene (**2**)
9	sucrose solution	sucrose solution + carotol (**3**)
10	sucrose solution	sucrose solution + daucol (**4**)
11	sucrose solution	sucrose solution + α-selinene (**5**)
12	sucrose solution	sucrose solution + β-selinene (**6**)

### Analysis of Carbohydrates

A modified protocol from Reiter et al.^61^ was followed with all details provided in Methods S1. Five replicates per line were analyzed. GC-MS data were transformed to .cdf or .mzML files and processed (ADAP chromatogram builder, chromatogram deconvolution, multivariate curve resolution) and aligned (ADAP aligner) with MZ Mine 2 (v 2.53).^62^ Differences in the amount of monosaccharides (including fructose and glucose), disaccharides (including sucrose), and total sugar between lines were tested. Following tests for data normality (Shapiro–Wilk) and equality of variances (Levene’s test), data were either analyzed using an ANOVA or a Kruskal–Wallis rank sum test. Since no absolute quantification was conducted, response factors for the various sugars were not corrected in the analysis.

### Analysis of Volatiles

As carrot flowers vary in size and weight, we decided not to focus on the absolute amounts of compounds (although, as sampled, the two sterile lines contained significantly less amounts of total volatiles compared to the male fertile lines LF and MF, and the OP line (OP H)) but instead focused on the differences in relative amounts between the lines. Due to the low nectar volumes in carrot flowers, there is no available method to separate the nectar from the remaining floral tissue and pollen without cross-contamination, making it practically impossible to treat nectar as a separate entity in the analyses. In nectar-rich flowers, extraction with microcapillaries, or other physical separation methods, can be used,^63^ while in nectar-poor flowers like carrots, we are limited to centrifugation, which is likely to also extract some sap with the nectar, or dipping, which will extract any compounds that are to some extent water-soluble from the surface of the flowers.

The floral volatile extracts in dichloromethane were analyzed with the same instrumental setup and method as for the carbohydrate analysis but without derivatization. Individual flowers were removed from the umbellets with scissors. Flowers from 3 umbellets were used per sample. The flowers were extracted with dichloromethane (500 μL) for 24 h in 2 mL vials, before the extracts were individually transferred to new vials. GC-MS injections (1 μL) were performed in splitless mode (1 min). Initially, these results were compared with previously reported results using headspace analysis with solid-phase microextraction (SPME).^64^ After confirming that the compound profiles were comparable (i.e., presence of monoterpenes and sesquiterpenes), five replicates of dichloromethane extracts per line were analyzed. Two samples were excluded from the data set due to failed extractions.

Differences in the total floral volatile amounts between lines were tested in two analyses: testing between all five separate lines and testing between three groups of lines. For this second analysis, the lines were pooled into three groups: low-yielding lines (LS and LF), medium-yielding lines (MS and MF), and OP high-yielding line H. Following tests for data normality (Shapiro-Wilk) and equality of variances (Levene’s test), data were either analyzed using an ANOVA followed by pairwise *t* tests, or a Kruskal–Wallis rank sum test followed by pairwise Mann–Whitney U tests where appropriate. A Holm correction was used in the pairwise analyses. As the GC-MS data contained zero values, data were fourth root transformed, centered, and scaled prior to multivariate analysis.^65,66^ To visualize differences between samples, a principal coordinate analysis was generated from a Euclidean distance matrix, using the packages “vegan”^67^ and “ape”^88^ in R v 3.5.1.^68^ Candidate compounds were tentatively identified by comparisons to mass spectral databases (Wiley 9, NIST17) and were purchased or isolated from commercially available essential oils; for details, please see Methods S1.

### Analysis of Minerals

Each nectar sample was accurately weighed and digested in 100 μL of concentrated nitric acid. The volume was made up to 5 mL with Milli-Q purified water. Five replicates per line were run. A blank was prepared in the same way. Standards for Ca, K, Mg, Na, and Sr were prepared from 1000 ppm standards (HPS, North Charleston, USA). An Agilent Technologies 5100 ICP-OES was used for the analysis, set to measure line intensities in axial mode to increase sensitivity. An ionization suppressant consisting of 0.5% w/v CsCl was used. The following emission lines were measured: Ca: 393.366, 396.847, and 422.673 nm; K: 766.491 and 769.897 nm; Mg: 279.553 and 280.270 nm; Na: 588.995 and 589.592 nm; and Sr: 407.771 and 421.552 nm. Data were checked for normality (Shapiro–Wilk normality test) and equality of variance (Levene’s test). To test for differences between lines, either an ANOVA followed by a pairwise *t* test or a Kruskal–Wallis rank sum test followed by a Mann–Whitney U test were conducted in R v 3.4.2.

## Results

In the first part of this study, which consisted of a set of preliminary qualitative experiments, the responses of free-flying honey bees to carrot nectar extracted from the four parental carrot accessions (2 reciprocal pairs) were investigated in field-based bioassays. The four accessions were selected based on available seed yield data (Figure 1A, data provided by Rijk Zwaan Pty Ltd., Australia). Two reciprocal pairs of male sterile CMS and fertile maintainer accessions were chosen as representative low-yielding (LS and LF) and medium-yielding (MS and MF) pairs. To determine the attractiveness of the nectars to honey bees, the nectar of flowers from each parent line was extracted and presented in feeders located in the vicinity of two full-size honey bee hives. These nectars failed to elicit any honey bee attraction even after several hours. To control for sugar content (and hence reward) between the samples, we next developed a set of experiments where equal volumes of carrot nectar were added to aqueous solutions of honey containing 40% sucrose. These solutions were assessed for honey bee attraction utilizing a dual-choice design with choices of either honey solution spiked with carrot nectar (treatment) or honey solution alone (control). In these experiments, with all four accessions, ≤2 bees were observed to feed on treatments, while, on average, >15 bees were observed to feed on controls. Two extraction methods, spinning (spun) and extraction with water (dipped), yielded comparable results (Figure 1B).

**Figure 2:**
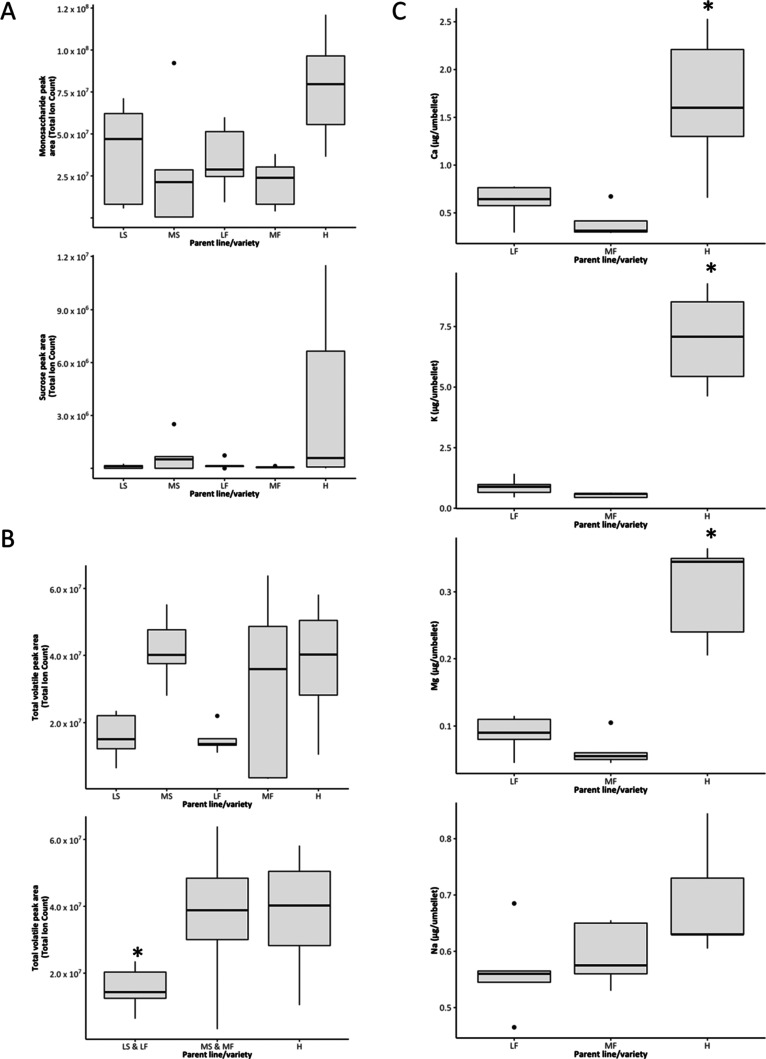
(A) Relative amounts of monosaccharides and sucrose in the sampled carrot accessions. (B) Relative amount of total volatiles (a) in the five carrot accessions, (b) in each of the three groups: low-yielding pair (LS and LF), medium-yielding pair (MS and MF), and OP H. (C) Content of Ca, K, Mg, and Na in parent lines LF and MF, and OP H (male sterile lines not analyzed). Boxes indicate interquartile ranges with the inner line denoting the median value.

These field-based experiments indicated that all four parental carrot accessions likely contained components unattractive to bees, as the bees approached but seldom landed on the treatment feeders, while they frequently landed and fed from the controls. Furthermore, those that did land on the treatments hesitated to taste and never consumed all of the solutions within the feeders, whereas those that landed on the control feeders consumed all (50 μL) of the honey solution. Guided by the initial field bioassay observations showing that carrot nectar deterred approaching bees from both landing and feeding on feeders, we designed a laboratory-based experiment combining both odor and taste factors. To facilitate quantitative experiments, a dual-choice bioassay in small cages was developed. Furthermore, a fifth high-yielding, OP cultivar, known to be relatively attractive to honey bees, Western Red (H), was included in the laboratory bioassay experiments. To rule out that no other factors of the honey solutions affected the feeding, plain sucrose solutions were used as controls instead of honey solutions in these experiments. Again, addition of carrot nectar from any of the four parental lines to sucrose solutions significantly reduced feeding (*P* < 0.001, *n* = 10, Figure 1C). The amount of nectar consumed by the bees showed a trend (Pearson correlation coefficient: *r*(3) = 0.85, *P* = 0.071) that corresponded to the differences in the reported seed yield for each line (i.e., bees consumed more nectar from lines that had higher seed yields). In a more detailed dual-choice experiment, sucrose solutions were spiked with nectar from OP H compared with sucrose solutions spiked with nectar from parent accession LF; bees consumed significantly less nectar from LF than from H (*P* = 0.031, Figure 1D). Based on these observations, nectar from the four parental accessions (LS, MS, LF, and MF) and the OP H cultivar were subjected to detailed chemical analysis to determine which chemical factors contributed to the lack of both initial attraction and continuous nectar feeding. Odorous repellents as well as minerals were targeted in the search for antifeedants. To determine any differences in potential nectar rewards, the levels of sugars in the nectar extracts from parent accessions LS, MS, LF, MF, and OP H were analyzed. Although slightly higher sugar concentrations were observed in H, no significant differences in the number of monosaccharides, disaccharides, or overall total sugars were found between the accessions (*P* > 0.05, Figure 2A).

AES analysis of nectar from all fertile varieties (LF, MF, and H) found that the H variety had a significantly greater amount of K and Mg than fertile parents LF and MF (Mann–Whitney U tests, *P* = 0.024 for all), and a significantly greater amount of Ca than MF (Mann–Whitney U test, *P* = 0.048). No significant difference in Na was observed (ANOVA, *P* = 0.07; Figure 2C). Male sterile lines were not analyzed.

To determine whether the nectars contained odorous repellents, we undertook GC-MS analysis of the organic profile of the aqueous nectar extracts by extracting the aqueous portion with an intermediately polar solvent, dichloromethane. Analysis of the organic extract indicated differences between the lines, although the extremely low concentrations of volatiles in carrot nectars made it difficult to quantify these differences. However, as has been reported for other plant species,^69^ organic extracts of whole flowers showed the same compounds were present in much higher quantities in our carrot floral samples, allowing multivariate quantitative analysis and comparisons between lines to pinpoint the differences in attraction. We used this methodology to assess 23 samples with 112 unique compounds detected.

Before investigating any specific compounds, the total amount of floral volatiles was analyzed, revealing that there was no significant difference in the amounts of total volatiles between each of the five carrots lines individually (Kruskal–Wallis rank sum test, *P* = 0.09, Figure 2B). When analyzed by male sterile/fertile pairs ((LS and LF) vs (MS and MF) vs H), a significant global difference in total volatile amounts between these groups was observed (*P* = 0.01, ANOVA) with the low yielding (LS and LF) having lower total volatile amounts than the medium-yielding (MS and MF; *P* = 0.03, pairwise *t* test) and high-yielding H (*P* = 0.02, pairwise *t* test, Figure 2B) carrot lines.

**Figure 3:**
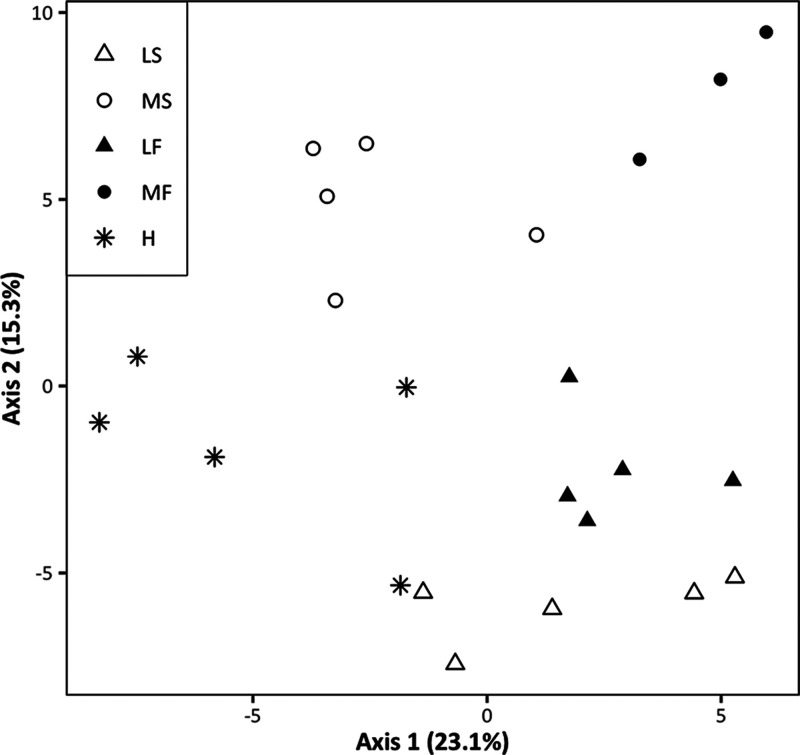
Principal coordinate analysis based on the abundance of 112 compounds detected in extracts from carrot accessions (LS, MS, LF, MF) and cultivar H. The relative corrected Eigen values denoting the percentage contribution of each axis to the total variation are displayed in the axes titles.

The principal coordinate analysis of the content of volatiles showed that the five carrot accessions had broadly overlapping clusters of compounds (Figure 3). Separation was observed along both the first and second axes. Fertile accessions LF and MF separated from male sterile LS and OP cultivar H along the first axis, with male sterile line MS occurring in the middle of these two groups. Line pair LS and LF separated from pair MS and MF along the second axis, with OP H occurring in the middle. Cumulatively, the first two axes contributed 38.4% of the total variation (axis 1:23.1%, axis 2:15.3%).

**Figure 4:**
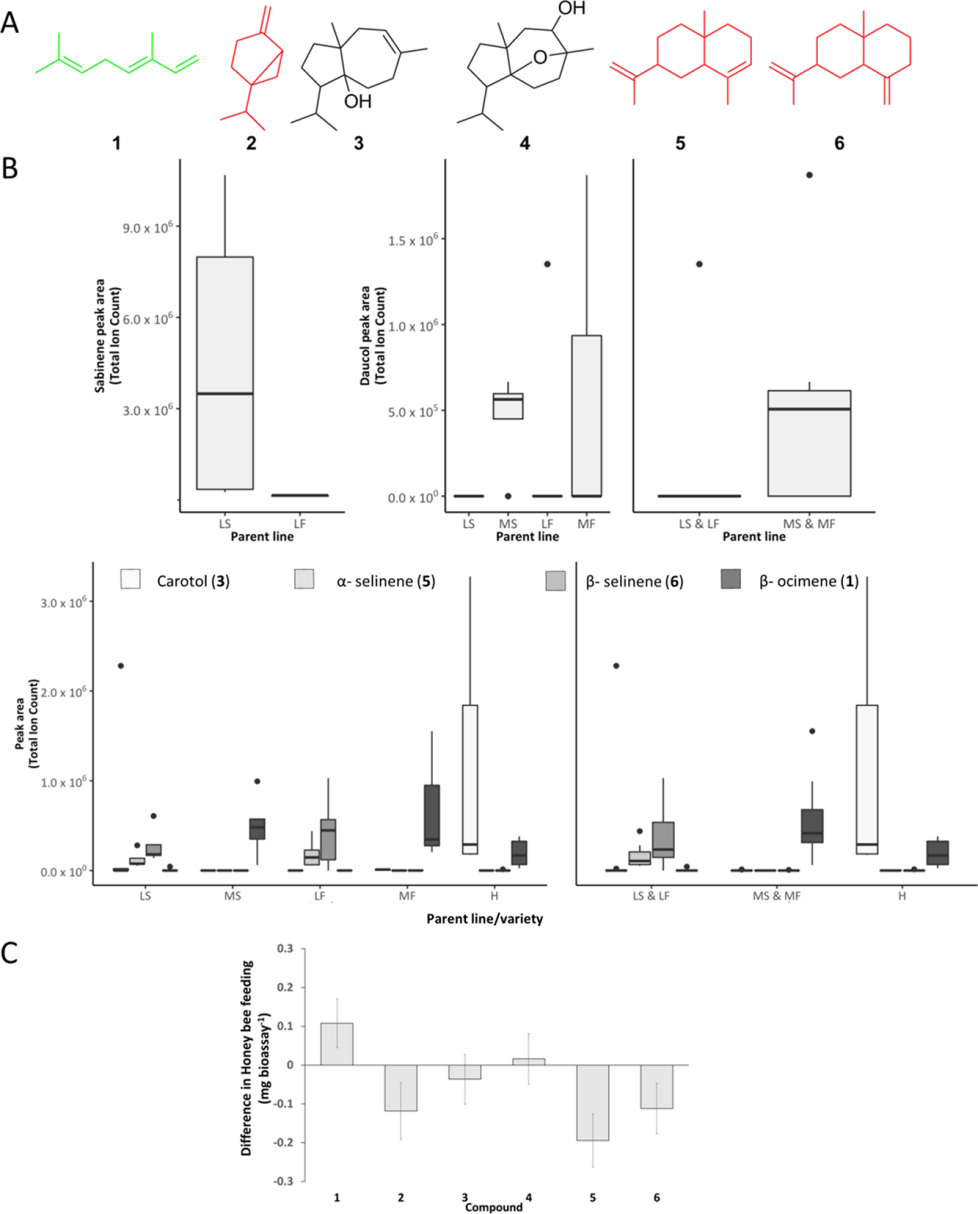
(A) Compounds identified from floral extracts and nectar extracts from carrot accessions LS, MS, LF, MF, and H: **1** = β-ocimene, **2** = sabinene, **3** = carotol, **4** = daucol, **5** = α-selinene, and **6** = β-selinene. Structures in red indicate reduced feeding, black neutral, and green increased feeding by honey bees when the respective compound was added to sucrose solutions in choice tests. (B) Occurrence of identified compounds in the carrot accessions (LS, MS, LF, MF, and H). (C) Laboratory bioassay results of pairwise choice trials with bees feeding on sucrose solution vs sucrose solutions spiked with synthetic compounds. Boxes indicate interquartile ranges with the inner line denoting the median value.

Compounds that showed differences in abundance by GC-MS analysis between or among any accessions and could be reliably identified were further investigated (Figure 4A). Primarily, compounds most abundant in the accessions with lowest recorded seed set were targeted as these compounds are more likely to be repellent. First, in the LS and LF pair, the monoterpene sabinene (**2**) was more abundant in the male sterile accession (LS) (*P* = 0.007, Mann–Whitney U test, Figure 4B) and was therefore considered a candidate honey bee repellent. Next, the sesquiterpene alcohol daucol (**4**) was identified as a potential attractant compound, as it was found to be less abundant in the low-yielding pair (LS and LF) than in the medium-yielding pair (MS and MF, *P* = 0.0044, Mann–Whitney U test). In the comparison between all accessions, including OP H, two sesquiterpenes; α-selinene (**5**) and β-selinene (**6**), were identified as potential repellent candidates, as these compounds were found to be more abundant in the lowest yielding lines (LS and LF) (Figure 4A) than in the medium-yielding lines (MS and MF, *P* < 0.001, *P* = 0.003, respectively), Mann–Whitney U test). Additionally, the monoterpene β-ocimene (**1**) was identified as a candidate attractant, as it was found to be less abundant in the low-yielding lines (LS and LF) than in the medium-yielding lines (MS and MF, *P* < 0.001, Mann–Whitney U test) and OP H variety (*P* = 0.002, Mann–Whitney U test, Figure 4A). Carotol (**3**) was also identified as a potential attractant compound due to its greater abundance in the OP H than in both the low-yielding (*P* = 0.013, Mann–Whitney U test) and medium-yielding parent lines (*P* = 0.008, Mann–Whitney U test) (Figure 4B). All candidate compounds were purchased or isolated from commercially available essential oils (identity confirmed by NMR analysis in combination with coinjections with extracts using GC-MS).

Candidate repellent and attractant compounds were tested in dual-choice laboratory bioassays, where bees could choose to feed from a pure sucrose solution (control) or a sucrose solution spiked with a candidate compound (0.1 mg, treatment). This amount of added candidate compound ensured that the sucrose solution was fully saturated with each candidate compound. After a 24 h period, bees consumed a greater volume of control sucrose solution compared to the sucrose solution spiked with the candidate repellents α-selinene (**5**) (*P* = 0.012), β-selinene (**6**) (*P* = 0.018), and sabinene (**2**), although this latter difference was not found to be significant (*P* = 0.06, Figure 4C). For β-ocimene (**1**), a candidate attractant, the bees consumed more of the spiked sucrose solution than they did of the control sucrose solution (*P* = 0.029), while for carotol (**3**) and daucol (**4**), there was no significant difference in the amount of the sucrose solutions consumed (*P* = 0.307 and *P* = 0.98, respectively).

## Discussion

In this study, we used a comprehensive approach applying methods from chemical ecology and pollination biology^70^ to identify specific traits affecting honey bee attraction to parental accessions and OP carrot flowers. Considering the need for cross-pollination, only traits present in both male sterile and fertile parent accessions, such as nectar and floral volatiles (but not pollen), were targeted.^57^ The results from our initial field bioassays suggested that the carrot nectars were not only lacking in attraction but also were in fact repelling bees from the feeders. These observations guided us to develop a new laboratory bioassay protocol, based on the concept by Kessler et al.,^71^ in which we were able to evaluate the combined effect of odor and taste. Results from these laboratory bioassays with nectar from each parental accession revealed a trend between a relative lack of honey bee attraction in the trials and recorded low seed yield per line. Further, there was a significant difference between the amount of nectar that bees consumed from the parental accession LF (low consumption) and the OP Western Red (H, high consumption) in these bioassays, again matching the known honey bee attraction to these lines. Chemical analyses of various plant types have demonstrated that nectars contain many more types of compounds than sugars and nutrients that provide pollinators with a floral reward. Indeed, nectars contain a suite of chemical compounds that can act as antifeedants, which can even be toxic to pollinators as shown in many studies, for example in ant- and honey bee pollination.^72−74^ Secondary metabolites, such as some phenolic compounds, iridoids, and alkaloids,^27,75,76^ carbohydrates, such as xylose,^77^ or inorganic elements, such as potassium,^78^ have been shown to deter pollinator visitation. Why secondary metabolites, including antifeedants and repellents, are incorporated in plant nectars is largely unknown (see Stevenson et al.^27^ and references within). In many plants, there is a strong correlation between the secondary metabolites in nectar and in other floral tissues, with the same compounds often present in nectar, but in lower quantities.^27^

In agreement with previous studies,^57^ we show that the sugar levels and composition in our samples varied greatly between individuals, but no significant difference between accessions was observed. Our results show that sugars and thereby reward quality were not linked to the observed differences in bee attraction between the accessions. In our AES analysis of minerals, we showed that the concentration of calcium, magnesium, and potassium differed significantly between the parental accessions and the OP Western Red (H) cultivar, despite being grown in the same soil. Notwithstanding these differences, the mineral contents were all more than 10 times lower than the levels previously reported as biologically significant in nectar from other crops, such as potassium levels in nonattractive onion^79^ and avocado^80^ nectars. Furthermore, the most attractive accession (H), contained the highest amount of the putatively repellent minerals (e.g., potassium), suggesting these do not constitute a major factor of the observed weak attraction to parental carrot nectars. For volatiles, the compound profiles were similar in both extracts of nectar and whole floral tissues. The compound profiles overall corresponded with those previously reported from other hybrid carrot lines with headspace sampling methods.^13^ All identified discriminatory compounds were terpenes or terpene alcohols, all of which are relatively nonpolar in nature. Despite the low polarity, these compounds were isolated from aqueous nectar solutions, which led to the design of a bioassay where a small amount of each semiochemical could be incorporated into the aqueous sucrose solutions. Taking advantage of the partial water-solubility of our secondary metabolites, the emulsion of an organic compound in an aqueous solution would represent a mimic of the natural flower, providing a slow release of odors (smell), while the bees are also exposed to the test compounds within the sugar solution (taste). Furthermore, this study relied on access to carrot accessions growing under the same controlled conditions. By collecting flowers at the same point of development, we were able to analyze highly homogeneous samples, suitable for GC-MS analysis and multivariate data treatment, allowing reliable identification of candidate bioactive compounds. The clear correlation between the presence or absence of compounds, selective feeding by bees naive to carrots in our laboratory bioassays and documented seed yield for each accession, indicate that we developed an effective bioassay allowing the identification of several confirmed repellent and/or antifeedant compounds from our samples.

Monoterpenes and sesquiterpenes, such as those identified in this study, are already known from previous pollination studies. β-Ocimene (**1**) has been suggested to be a pollinator attractant^81^ and has been reported as a main constituent of the bouquet of floral volatiles emitted by wild parsnip (*Pastinaca sativa*), a basal relative of cultivated carrot.^82^ This compound is also a brood pheromone, regulating foraging in honey bees.^83,84^ Remarkably, despite the many suggested roles for β-ocimene in plant-insect systems,^81^ our study is the first to confirm this compound to be a floral attractant to honey bees. It is also important to note that by adding this compound to the same sucrose solution matrix as the repellent compounds, the mixture becomes more attractive to the honey bees, serving as a control of the bioassay design. Similarly, it has been shown in a study on Asteraceae that floral odor bouquets spiked with sabinene (**2**) as part of a more complex mixture, reduced honey bee attraction.^85^ For sesquiterpenes, it has been reported that β-caryophyllene and β-elemene are attractive to *Apis cerana*,^86^ while β-*trans*-bergamotene is believed to have an attractant effect on bumble bees.^87^ Terpenoids are also well-known antifeedants (herbivore defense) and antimicrobials (pathogen defense).^88^ Thus, from an evolutionary perspective, there is likely to be a trade-off between seed set and being eaten or infected. For example, the key repellent compound found in our study, β-selinene (**6**), is a known antifungal compound in the roots of maize, and also induced by jasmonic acid in celery.^89,90^ Furthermore, previous studies on *Brassica rapa* have shown that the evolution of most floral traits is affected by insect pollination and herbivory, showcasing the importance of these interactions for plant evolution.^91^ For breeding purposes, it would be fundamental to monitor and attempt to optimize the balance between pollinator attraction and plant defense. It may be suggested that rapid anthropogenic environmental change and artificial selection in cropping systems could have disrupted this balance, which should be addressed in future breeding programs.

In conclusion, we unambiguously show that individual compounds isolated from carrot nectar and floral extracts directly impact feeding of honey bees in behavioral bioassays and subsequently may impact pollinator visitation in carrot seed production. This finding is a key step toward the development of targeted plant breeding methods for the design of hybrid carrot seed crops with improved pollinator attraction and seed yield. Plant breeding programs can now target the reduction of the levels of the identified repellent terpenes β-sabinene, α-selinene, and β-selinene^92^ to improve pollination rates in these crops for increased seed production volumes. Furthermore, our developed methodology implementing chemical phenotyping of pollination semiochemicals employing GC-MS can be applied to identify traits to be modified for improved pollination efficiency in insect-pollinated crops with low seed yields generally.

## Supporting Information Available

Detailed chemical procedures, field bioassay and laboratory bioassay setup, and NMR data (PDF)
