# Supplementary material for: Critical Pollination Chemistry: Specific Sesquiterpene Floral Volatiles in Carrot Inhibit Honey Bee Feeding
Source: J Agric Food Chem. 2023 Oct 23;71(43):16079–89. doi: 10.1021/acs.jafc.3c03392 (PMC10623568; doi:10.1021/acs.jafc.3c03392)

Critical pollination chemistry: Specific sesquiterpene floral volatiles in carrot inhibit honey bee feeding

Stephen R. Quarrell, Alyssa M. Weinstein, Lea Hannah, Nicole Bonavia, Oscar del Borrello, Gavin R. Flematti and Björn Bohman

Methods S1 Detailed chemical procedures, field bioassay and laboratory bioassay setup, and NMR data

Analysis of carbohydrates

Carrot nectar extract (10 µL) and ribitol solution (20 µL, 0.2 mg/mL in H_2O) were mixed in a 2-mL glass vial, and evaporated to dryness under a stream of nitrogen gas at 37 °C. Methoxyamine HCl (20 µL of 20 mg/mL solution in pyridine; Sigma-Aldrich, St Louis, MI, USA) was added and the sealed vials were heated for 2 hours in a heating block at 37 °C. At the same temperature, the extracts were subsequently treated with *N*- methyl-*N*-(trimethylsilyl) trifluoroacetamide (MSTFA, 35 µL; Sigma-Aldrich, St Louis, MI, USA) in the same sealed vials for 1 hour before gas chromatography-mass spectrometry (GC-MS) analysis. GC-MS analysis was performed on an Agilent 5973 mass selective detector connected to an Agilent 6890 GC equipped with a BPX5 column [(5% phenyl polysilphenylene-siloxane), 30 m × 0.25 mm × 0.25 µm film thickness; SGE Australia], using helium as the carrier gas. An Agilent 7683 autoinjector was used and injections (3 µL) were performed in split mode (1 to 10). The GC oven was programmed: 40 (5 min), 5/200, 10/280(5 min). Injector and transfer line temperature: 280 °C. Mass spectra were recorded from *m/z*: 33-300. Tentative identification of trimethylsilylated monosaccharides and sucrose was based on the comparison of retention index and mass spectra with data from a mass spectral library (NIST14). All tentative identifications were confirmed by co-injections with synthetic standards (Sigma Aldrich, Australia).

Identification of volatiles

Ocimene (mixture of isomers, [13877-91-3]) was purchased from Sigma Aldrich, Australia and Sabinene [3387-41-5] was purchased from ChemExpress, China. The purchased standards were confirmed as >97% pure by GC-MS. Carotol and daucol were isolated from carrot seed oil (Range Products, Australia) by flash chromatography on silica gel (40 µm, 80 g, Buchi Reveleris X2 Flash Chromatography module) using a gradient elution of hexanes to ethyl acetate, flow rate 60 mL/min over 20 min. Selinene (α and β-isomers) were isolated from celery seed oil (Range Products, Australia) by a combination of flash chromatography on silica gel (as for carrot seed oil above, eluent: hexanes), Kugerrohr distillation (collected remaining residue after removing volatile fraction at 125 °C) and preparative HPLC using a Gilson 321 semi-preparative pump with a Gilson 215 liquid handler connected to an Agilent 1100 diode-array detector. Separation was achieved using a C18 reversed phase column (250 x 22 mm Apollo C18, Grace Discovery Sciences, Victoria, Australia) using an isocratic elution 85% (v/v) acetonitrile/water. Injection volume: 500 µL, flow rate 20 mL/min, 12 injections, gave α-selinene (4 mg) and β-selinene (50 mg) as separate fractions. All isolated natural products were structurally confirmed by ^{1}H NMR, ^{13}C NMR and GC-MS.

Field bioassays and laboratory bioassay setup

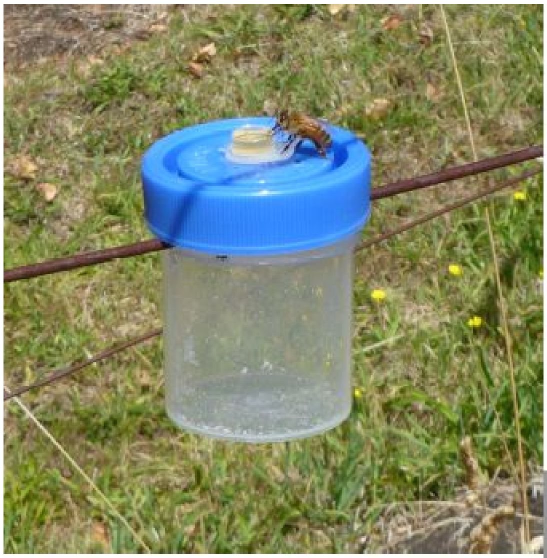

Left: Honey bee feeder used in field bioassays

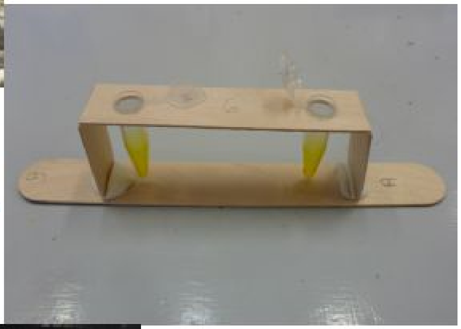

Right: Honey bee feeder used in laboratory bioassays

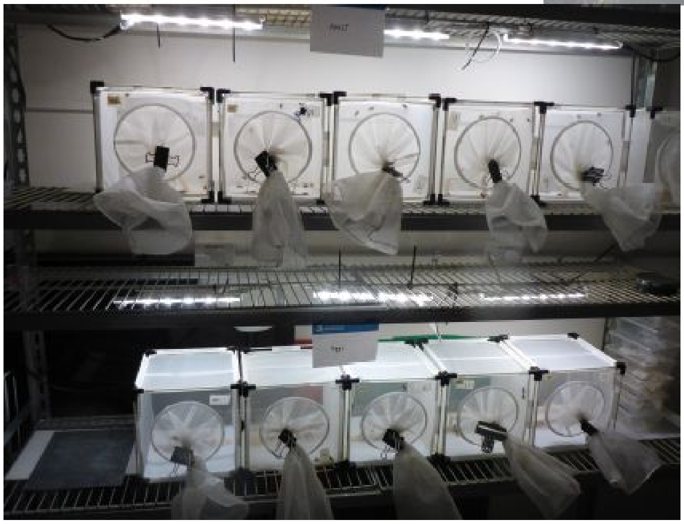

Left: Cages used for honey bee feeder used in field bioassays

^{1}H NMR α-selinene (**5**)

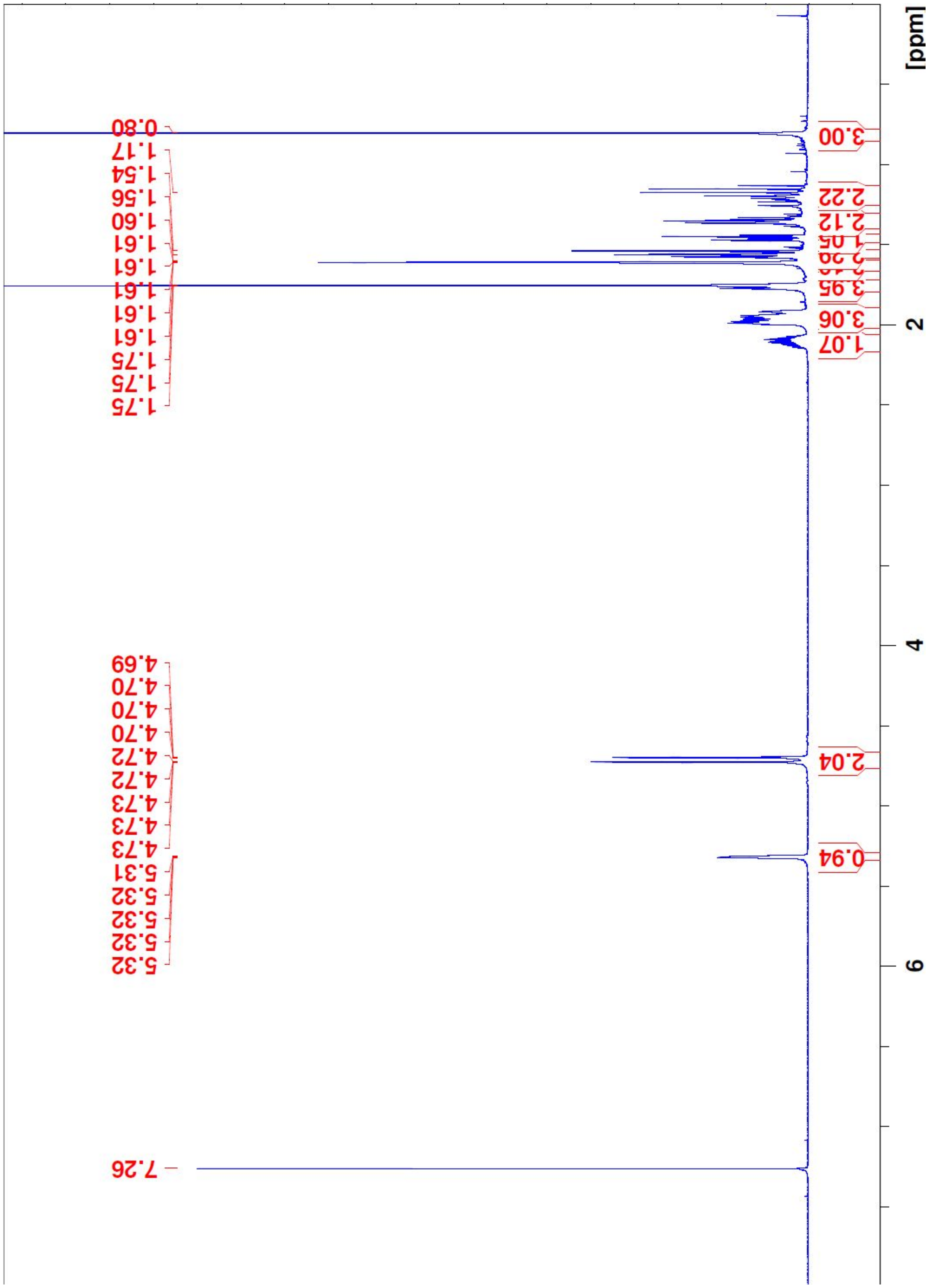

^{13}C NMR α-selinene (**5**)

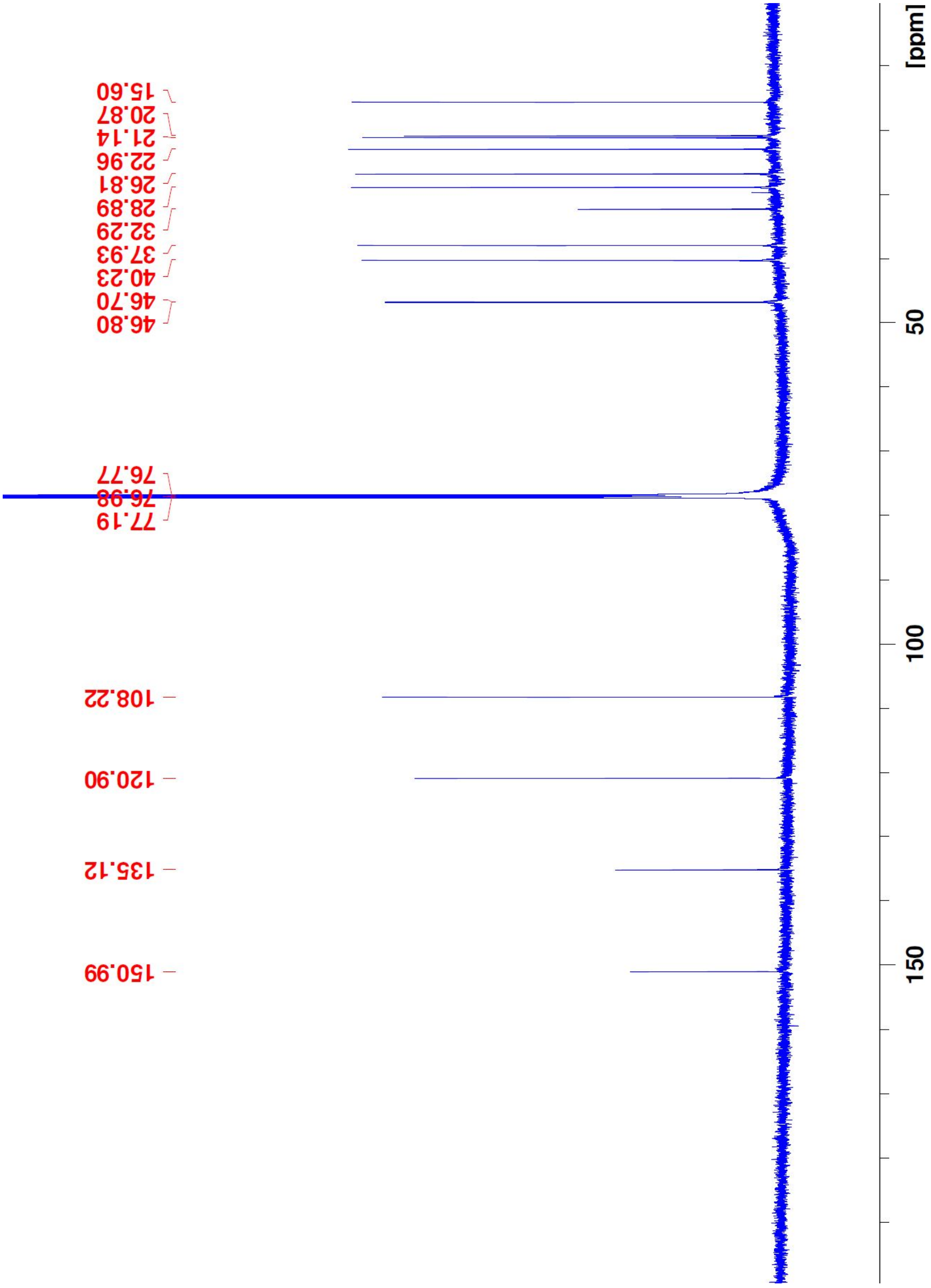

^{1}H NMR β-selinene (**6**)

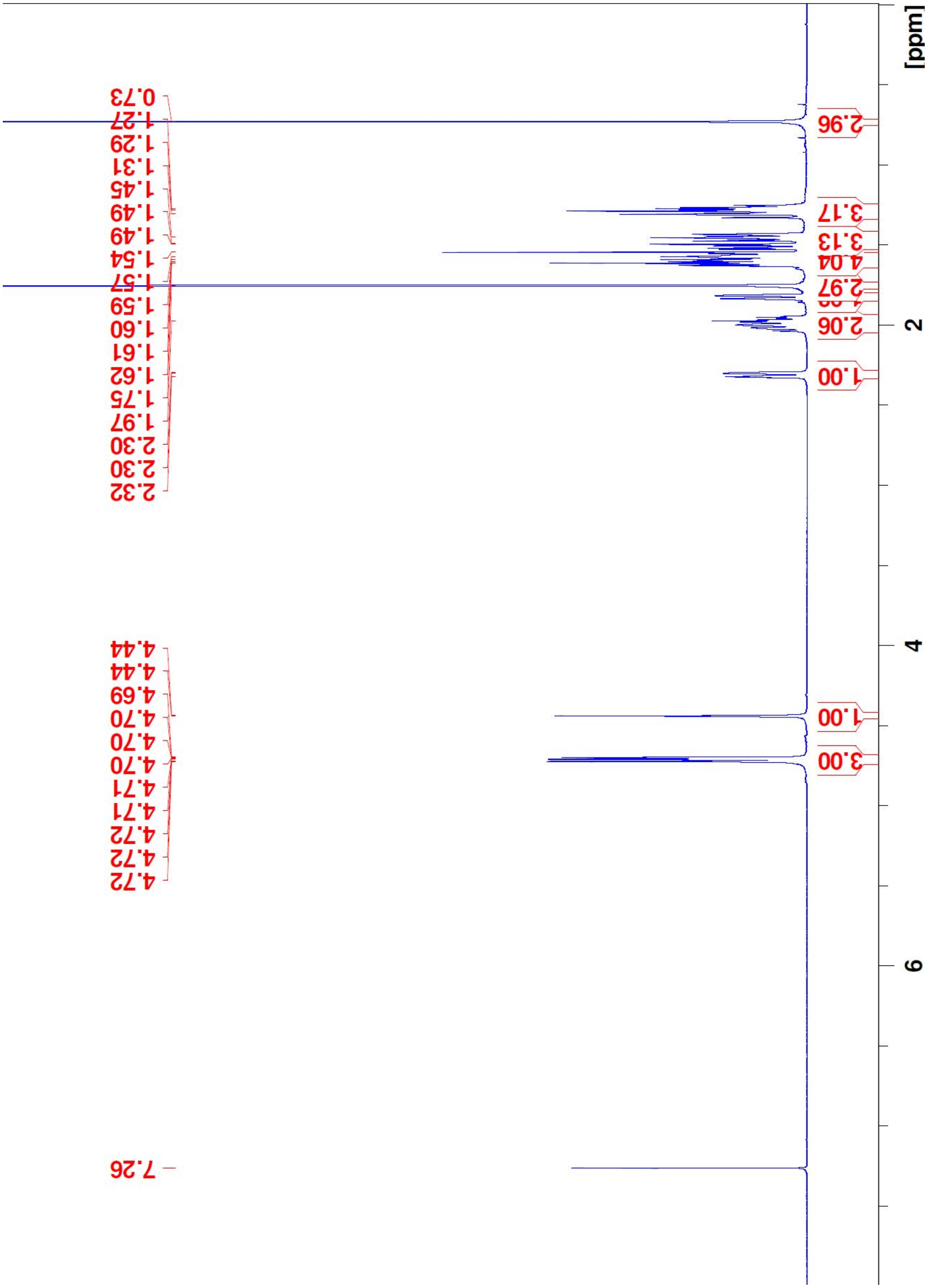

^{13}C NMR β-selinene (**6**)

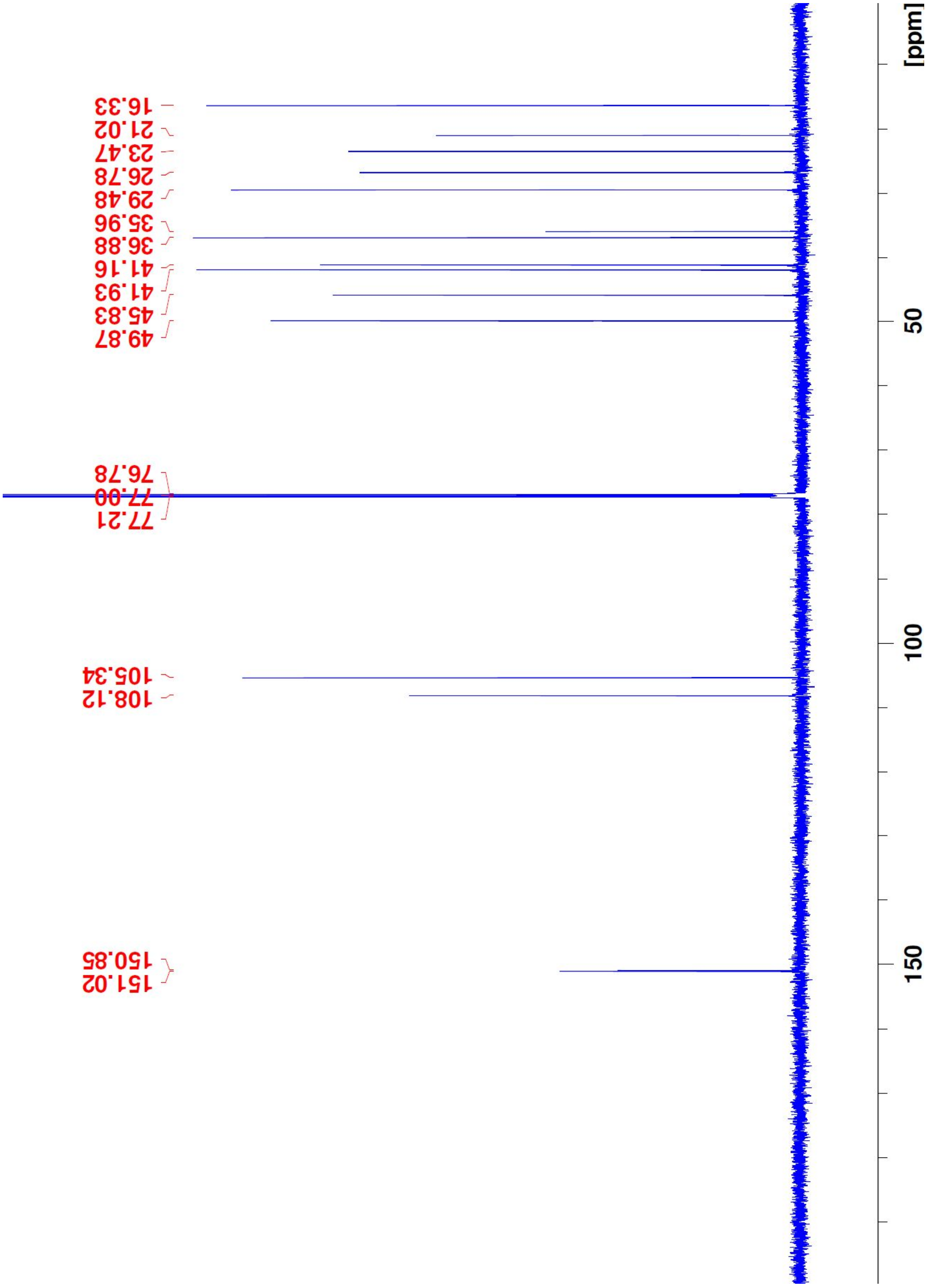

Supplement: Supplementary file 1 — jf3c03392_si_001.pdf [file jf3c03392_si_001.pdf]
